# A multiplexed nanostructure-initiator mass spectrometry (NIMS) assay for simultaneously detecting glycosyl hydrolase and lignin modifying enzyme activities

**DOI:** 10.1038/s41598-021-91181-8

**Published:** 2021-06-03

**Authors:** Nicole Ing, Kai Deng, Yan Chen, Martina Aulitto, Jennifer W. Gin, Thanh Le Mai Pham, Christopher J. Petzold, Steve W. Singer, Benjamin Bowen, Kenneth L. Sale, Blake A. Simmons, Anup K. Singh, Paul D. Adams, Trent R. Northen

**Affiliations:** 1 Joint BioEnergy Institute, Emeryville, CA 94608 USA; 2 Sandia National Laboratories, Livermore, CA 94551 USA; 3 Lawrence Berkeley National Laboratory, Berkeley, CA 94720 USA; 4 University of California, Berkeley, CA 94720 USA

**Keywords:** Biotechnology, Analytical biochemistry, Biochemistry, Carbohydrates, Enzyme mechanisms, Enzymes

## Abstract

Lignocellulosic biomass is composed of three major biopolymers: cellulose, hemicellulose and lignin. Analytical tools capable of quickly detecting both glycan and lignin deconstruction are needed to support the development and characterization of efficient enzymes/enzyme cocktails. Previously we have described nanostructure-initiator mass spectrometry-based assays for the analysis of glycosyl hydrolase and most recently an assay for lignin modifying enzymes. Here we integrate these two assays into a single multiplexed assay against both classes of enzymes and use it to characterize crude commercial enzyme mixtures. Application of our multiplexed platform based on nanostructure-initiator mass spectrometry enabled us to characterize crude mixtures of laccase enzymes from fungi *Agaricus bisporus* (*Ab*) and *Myceliopthora thermophila* (*Mt*) revealing activity on both carbohydrate and aromatic substrates. Using time-series analysis we determined that crude laccase from *Ab* has the higher GH activity and that laccase from *Mt* has the higher activity against our lignin model compound. Inhibitor studies showed a significant reduction in Mt GH activity under low oxygen conditions and increased activities in the presence of vanillin (common GH inhibitor). Ultimately, this assay can help to discover mixtures of enzymes that could be incorporated into biomass pretreatments to deconstruct diverse components of lignocellulosic biomass.

## Introduction

There is a growing need to find renewable and eco-friendly sources of energy. Lignocellulosic biomass has great potential to create sustainable drop-in replacements for conventional petroleum-based fuels. It is composed of three major biopolymers: cellulose, hemicellulose and lignin and therefore maximal breakdown will require efficient deconstruction of both the glycans via glycosyl hydrolases (GHs) and lignin by lignin modifying enzymes (LMEs)^1, 2^. It is desirable to develop chemically resolved assays that are suitable for simultaneously characterizing both GH and LME activities because lignin products can impede enzymatic saccharification through nonproductive binding interactions with carbohydrate-active enzymes^3, 4^. Finding enzymes/enzyme cocktails which can cleave both carbohydrate and phenolic bonds has great potential to help prevent this deactivation and aid in both lignin and glycan deconstruction towards the development of bioproducts.

Previously we have reported a range of chemically resolved nanostructure-initiator mass spectrometry (NIMS)-based glycoside hydrolase activity assays^5, 6^. NIMS is a surface-based mass spectrometry surface using a perfluorinated oil trapped in a nanostructured surface for direct desorption/ionization of compounds deposited on the surface^7, 8^. Its advantage over conventional techniques such as MALDI is that it does not require any organic matrix and therefore can resolve small molecules which are normally obscured by matrix background signals. NIMS enzyme assays use fluorous-tagged substrates that are strongly ionizing and adsorb onto the NIMS surface via fluorous phase associations, providing high signal-to-noise ratios for NIMS-tagged substrates and products. Most recently we expanded these assays beyond GH to characterize peroxidase enzymes that can cleave the β-O-4 linkage of lignin, but this platform technology can be used to examine the activities of other lignin-active enzymes, as well. Compared with popular assay method like colorimetric assay (e.g. ABTS assay for laccases, DNS assays for GHs) which provide information on the total extent of a reation, our mass spectrometry-based assay provide information on substrate specificity, product distributions, and enzyme kinetics^9, 10^. In addition, these assays can be performed with nanoliter volumes^11^ of samples and multiple substrates^12^ enabling simultaneous tracking of multiple reactions^13^.

Laccases are a class of lignin-active enzymes which catalyze single-electron oxidations of aromatic substrates by using oxygen as a terminal electron acceptor. When coupled with a mediator such as 2,2’-azinobis(3-ethylbenzathiazoline-6-sulfonate) (ABTS), laccases can oxidize nonphenolic substrates as well, making them a target enzyme for lignin deconstruction^14, 15^. To our knowledge no specific analytical methods have been developed to simultaneously characterize the breakdown product distribution of lignin and cellulose model compounds.

**Figure 1:**
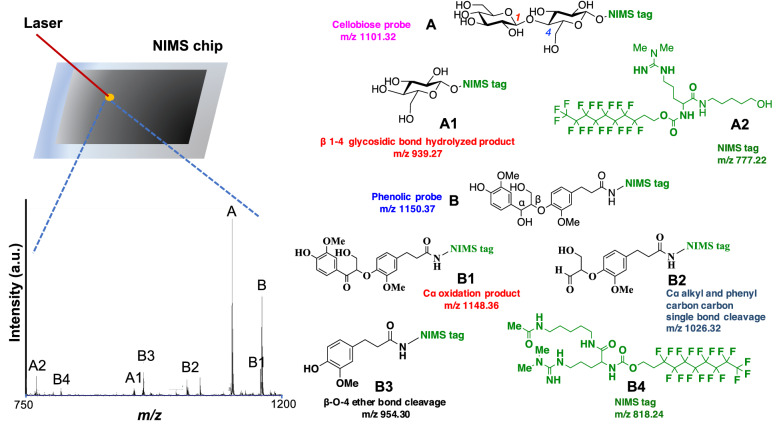
Concept of a NIMS multiplexed glycoside hydrolase and lignin modifying enzyme assay.

Here, we report the integration of existing NIMS assays using cellobiose probes^12^ with the recently reported phenolic β-aryl ether probe^16^ to simultaneously assay activities against lignin and glycan model compounds (Fig. 1). We selected the β-aryl ether bond for our studies because it is the most common linkage in lignin^17^. Laccase^18^ is an important class of enzyme that plays a key role in lignin degradation. In order to better understand the degrading efficiency of various laccases from different sources, we utilized our multiplexing assay to characterize two laccases from Agaricus bisporus (Ab) and Myceliopthora thermophila (Mt). We find that these two commercially-available fungal laccase samples which are known to contain mixtures of enzymes produced by the host fungi^19^ exhibit activities against both the glycan and phenolic probes to produce various products including “tag only products” (A2 and B4 in Fig. 1). We then use the multiplexed assay to characterize GH enzyme inhibition by the aromatics.

## Results

### Analysis of cellulases and hemicellulases activities

**Figure 2:**
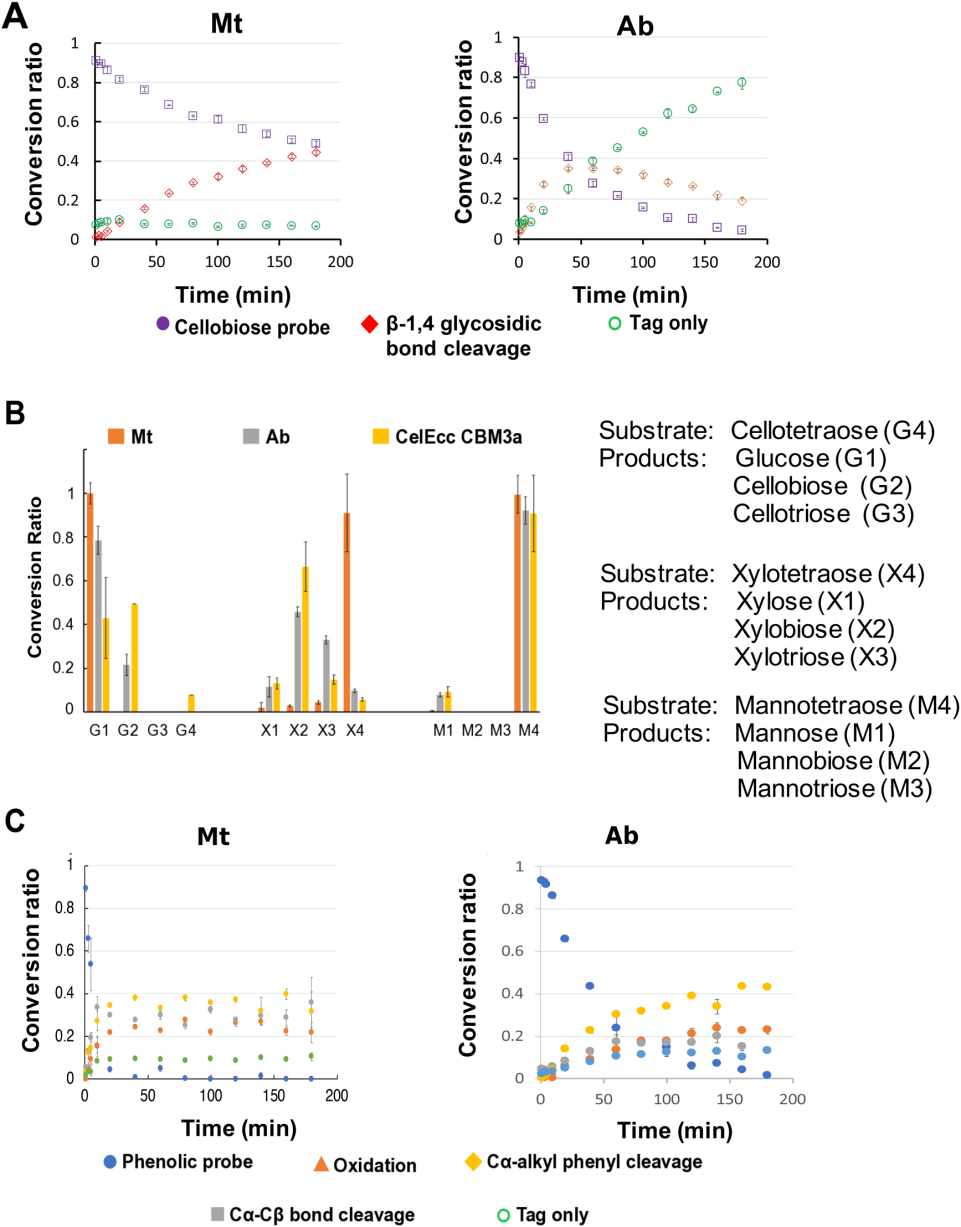
Independent analysis of glycoside hydrolase and lignin modifying enzyme activities Conversion of (**A**) GH probe *(n* = *3)* and (**C**) LME probe *(n* = *3*)*.* For both assays, **e**nzyme loading concentrations were 0.74 μg and 0.15 μg per 0.83 mM probe for Mt and Ab, respectively. The conversion ratio is expressed as the relative intensity of product and substrate peaks. (**B**) Conversion of oxime-NIMS-tagged oligosaccharides (n = 3). Enzyme loading concentrations were 1.4 μg and 0.3 μg per 1 mM xylotetraose or cellotetraose for Mt and Ab, respectively. The conversion ratio is expressed as the mass ratio of products, determined by normalization to C13 internal standards.

We first tested the two commercial laccase samples from *Agaricus bisporus* (*Ab*) (Sigma 40452) and *Myceliopthora thermophila* (*Mt*) (Novozymes, Novozym 51003). For brevity, the laccase samples, used without further purification, will be referred to as Ab and Mt, respectively. Since both are crude over expression preparations of fungal laccases and we expected would have GH activities from endogenous cellulases^20^. To confirm the presence of GH activities, we first tested their activities against our GH probe using a time series analysis (Fig. 2a) and observe 50% conversion in approximately 3 h. For a more detailed analysis of the GH activities we used unmodified cellotetraose, xylotetraose and mannotetraose as substrates and performed post-enzymatic NIMS tag derivatization for analysis. For comparison, we included a purified multifunctional CelEcc-CBM3a which we have previously characterized in detail^21^ and has strong activity against all three substrates. As expected, we observe high levels of activity against all three substrates from the positive control (Fig. 2b). In addition, we find that both commercial mixtures exhibited activity towards both cellotetraose and xylotetraose, reflecting relevant cellulase and hemicellulase activities. However, only Ab exhibited some degree of activity towards mannotetraose whereas Mt had lower activity than Ab towards xylotetraose. The observed activities towards the cellobiose NIMS probes and purified oligosaccharides are attributed to the GH enzymes (cellulase, xylanases, or mannanases) that are produced by the two fungal hosts, confirming the suitability of these two commercial mixtures for development and application of the desired multiplexed analysis of GH and LME activities.

### Analysis of lignin modifying enzyme activities

The same time series analysis was performed using Ab and Mt against the β-O-4 phenolic probe. To provide a basis for comparing the laccase activities in the two laccase enzymes, we performed a standard ABTS colorimetric assay (Sigma Aldrich, Roche ABTS) and normalized the enzyme dilutions to the same ABTS activities. As shown in Fig. 2c, we observe cleavage of the phenolic probe by both enzyme mixtures. Ab shows strong activity with > 90% cleavage within the first 10 min. By contrast Mt requires approximately 120 min to reach this level. Both enzyme mixtures produce all four breakdown products: oxidation (B1), Cα-alkyl phenyl cleavage (B2), Cα-Cβ bond cleavage (B3), and cleavage from the probe from the NIMS tag (B4).

### Multiplexed cellobiose (Fig. 3A) and phenolic (Fig. 3B) probe assay

**Figure 3:**
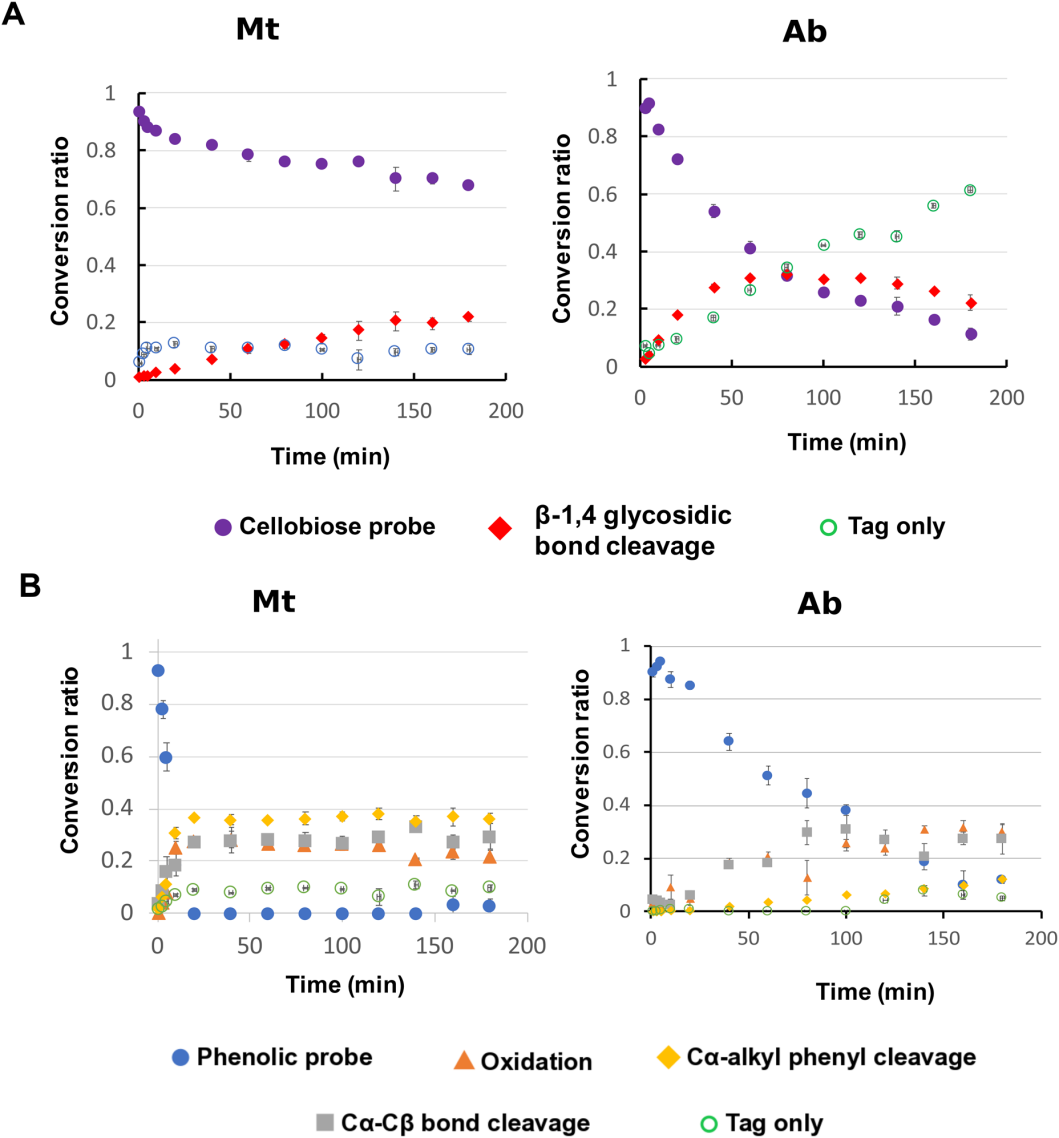
Multiplex assays against mixed GH and LME probes *(n* = *3).* (**A**) Conversion of GH probe in the presence of LME probe. (**B**) Conversion of LME probe in the presence of GH probe. Enzyme loading concentrations were 0.74 μg and 0.15 μg per 1.66 mM total substrate (0.83 mM GH probe and 0.83 mM LME probe) for Mt and Ab, respectively.

Having independently confirmed the activities of the two commercial enzyme mixtures against both GH and LME substrates we then used the mixed substrate assay conceptualized in Fig. 1. In this first test of the integrated NIMS GH and LME assay, we characterized both ABTS-normalized Mt and Ab samples against a mixture of the β-O-4 phenolic probe and the cellobiose probe using a time-series analysis (Fig. 3). As expected, given the independent GH and LME assays (Fig. 2) we found that both exhibited activity towards the GH and LME probes in the mixed assay. While the LME activities were similar to those detected in the single substrate assay, we observe reduction in the GH activities, which are attributed to the anticipated GH inhibition by aromatic products. At the 3 h point, Ab conversion of the GH probe was approximately 17% lower in the presence of LME (82.1 ± 0.8% conversion of the GH probe as a single substrate and 68.9 ± 3.7% conversion of GH probe in the presence of LME probe). Similarly, at the 3 h point, Mt conversion of the GH probe was approximately 19% lower in the presence of LME LME (51.2 ± 1.2% conversion of the GH probe as a single substrate and 32.2 ± 0.8% conversion of GH probe in the presence of LME probe).

### Enzyme inhibition studies

**Figure 4:**
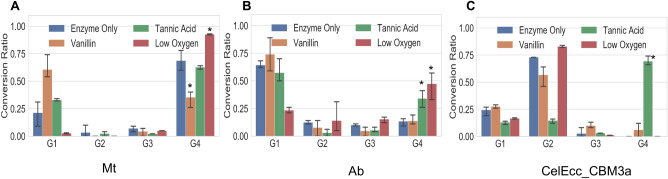
Effect of mediators and inhibitory conditions on cellotetraose degradation by (**A**) Mt (**B**) Ab and (**C**) CelE CBM3a *(n* = *3)*. “*” indicate ANOVA post hoc significant differences compared to enzyme only. Enzyme loading concentrations were 1.4 μg and 0.3 μg per 1 mM cellotetraose for Mt and Ab, respectively. (G1 = glucose; G2 = cellobiose; G3 = cellotriose; G4 = cellotetraose) Inhibition reaction was run at 37 °C for 20 min; Oxime tagging reaction time = 22 h.

To further investigate enzyme inhibition using our multiplexed assay we focused on the known GH inhibitors vanillin^22–26^ and tannic acid^27–29^ (Fig. 4) and included CelEcc-CBM3a as a control. To limit activity from laccase, and potentially other oxidative enzymes which may be present in the laccase samples, such as LMPOs, we included a low-oxygen condition using an anaerobic chamber N_2_-sparged buffer. We find that in the presence of vanillin, only CelEcc-CBM3a (an endoglucanase) showed a statistically significant decrease in cellotetraose conversion, whereas the laccase mixtures showed an increase in cellotetraose conversion. Under oxygen-limiting environments both Ab and Mt showed diminished activity towards cellotetraose, consistent with what has been reported for LPMO^30^ enzymes, whereas CelEcc_CBM3a performance was not affected.

## Discussion

Previously, we have developed probes to target stereospecific bond cleavage in glycan^12^ and lignin substrates^16^ by cellulases and lignin modifying enzymes, respectively. By combining these two probes in a multiplexed assay, we have developed a platform that can rapidly screen for enzymes/cocktails capable of degrading both lignin and glycan substrates and examine enzyme inhibition.

As shown in Fig. 2, both commercial enzyme mixtures were capable of cleaving cellobiose, with Ab showing the fastest cleavage, indicating the presence of desired GH activities endogenous to the host. Both showed activities against cellotetraose and xylotetraose GH substrates. Overall, Ab demonstrated higher degree of cellotetraose degradation than Mt under the same pH and temperature conditions used for the GH probe assay. We also observed the anticipate activities against the phenolic probe, consistent with literature^16^ and well-documented laccase activities towards phenolic substrates. Overall, the Mt mixtures showed higher activity against the phenolic probe and Ab has higher activities against the GH probe. However, we observe diminished activities vs. what was observed using the purified substrates indicating the anticipated inhibition of the GHs by the phenolic products. This reflects a very relevant problem in development of enzyme cocktails for lignocellulose deconstruction^31^ since enzyme treatments represent a major cost in most biomass to bioenergy processes, and the longer reaction times and increased enzyme loadings required to compensate for enzyme inhibition are relevant to the overall viability of the process^32^.

While analysis of model compounds provides important information on substrate specificity and products that is difficult to interpret from analysis of complex biomass mixtures, it is well-known that screening of model substrates often fails to translate to real biomass feedstocks^33^. Therefore, we also tested Ab and Mt over AFEX pretreated switchgrass. The results (shown in the supplementary Fig. 1) confirmed the celluases and hemicellulases activities in Ab and Mt samples.

To further examine enzyme inhibition using our multiplexed assay we selected two known inhibitors of GHs tannic acid and vanillin. Since the use of model compounds can introduce potential bias due to the long fluorinated tags, we used tetramers as our substrates for enzyme inhibition studies. A chemical probe^9^ was utilized to quantify glycan products following the enzymatic reactions. In addition, since it is possible that LMEs may contribute directly to glycan cleavage we also explored low oxygen conditions. Aromatics are thought to inhibit cellulases and xylanases by nonproductive binding^23^ and we found clear inhibition of activities of Ab and CelEcc-CBM3a by tannic acid (Fig. 4, green color bars), but not for Mt. Interestingly we observe clear inhibition of CelE-Ecc3a by vanillin (peach color bars) and we observed increase activities against cellotetraose by Mt. Since vanillin can act as a mediator for radical enzymes such as laccases and LPMOs, helping the single electron transfer between laccases and the substrates^32, 34, 35^, this suggested some direct contribution of the LMEs to GH cleavage. Consistent with this view, we also observed inhibition of GH activities under low-oxygen conditions. Specifically, both Mt and Ab showed a statistically significant decrease in activity towards cellotetraose when reactions were under oxygen-limiting conditions (*p* < 0.001), which would be expected for an oxygen dependent mechanism and not for hydrolytic GH enzymes such as CelEcc_CBM3a which was unaffected. Since fungi are known to produce LPMOs and these enzymes are known to cleave GHs^36^, one explanation is that these commercial enzyme mixtures contain LPMOs. Another possibility is that laccases may themselves act directly on GHs, though we see no literature precedent for this. Further investigation of the possible GH activities of laccases would require purified enzymes which are unfortunately not commercially available.

## Conclusions

In this work, we demonstrated a multiplexed NIMS-based assay which enables characterization analysis of commercial laccase enzyme mixtures against lignin and glycan model compounds. These assays enable simultaneous analyses of both glycoside hydrolase and lignin modifying enzyme activities which we anticipate will be helpful in developing enzyme cocktails for lignocellulose deconstruction. Importantly, we find that our assay is suitable to examine the inhibition of GH activities by aromatics which is an important consideration in the development of economically viable biomass to biofuels approaches.

## Experimental section

### Materials and methods

Laccases *Agaricus bisporus* (product no. 40452) and *Myceliopthora thermophila* (product Novozyme 51003) were purchased from Sigma Aldrich. Prior to use, each laccase was either reconstituted or diluted in ultrapure water and activity was determined by oxidation of 0.2 mM ABTS (Sigma Aldrich, Roche ABTS solution), monitored at 420 nm (ε_420_ = 3.6 × 10^4^ M^−1^ cm^−1^) over the course of 15 min. Laccase stock solutions used subsequently in experiments were normalized to equivalent activities. CelEcc_CBM3a was provided by Brian Fox (Great Lakes Bioenergy Research Center, Madison, WI, USA) and prepared as a 6.5 mg/mL stock in ultrapure water. Enzymes were used without further purification. To confirm the presence of carbohydrate-active enzymes in laccase samples, enzymes were submitted for proteomics analysis (The mass spectrometry proteomics data have been deposited to the ProteomeXchange Consortium via the PRIDE^37^ partner repository with the dataset identifier PXD020215 and 10. 6019/PXD020215). Proteomics data to confirm the presence of laccases and GH enzymes is presented in the supplementary information (table 1 and 2). Protein concentrations of enzyme solutions were determined using a modified Lowry assay (Bio-Rad *DC* Protein Assay; Bio Rad Laboratories).

For the NIMS assays, 5 μL of enzyme diluted or reconstituted in ultrapure water (or ultrapure water for controls) was added to 5 μL of 100 mM sodium acetate buffer, pH 4.6 in 0.2 mL PCR tube. 1 μL of 10 mM cellobiose (GH) probe and 1 μL of 10 mM phenolic β-O-4 (LME) probe water were subsequently added to the mixture, vortexed with a Vortex mixer and incubated in a Thermo mixer (37 °C, 700 rpm). For samples without one or both probes, an equivalent volume of ultrapure water was used. At each assay time point, a 0.2 μL aliquot of the reaction sample was directly spotted onto the NIMS surface and removed after 3 s by wicking with a kimwipe. Enzyme reactions for the three assay conditions (GH probe + LME probe, LME probe only, and GH probe only) were run in triplicate.

Soluble oligosaccharide studies were performed using cellotetraose, mannotetraose, and xylotetraose (Megazaymes). Reducing sugars were derivatized for NIMS detection by post-enzymatic reaction using oxime tagging techniques^9^. [*U*]-^13^C glucose and [*U*]-^13^C cellobiose (Omicron Biochemicals)were included in the derivitazation process and used as internal standards for oligosaccharide experiments. Oxime tag synthesis and oxime tagging with internal standards was performed as discussed previously^9^.

For inhibitor, mediator, and control experiments 10 μL of enzyme was added to 10 μL of buffer (100 mM sodium acetate buffer pH 4.6 for laccases and 100 mM sodium phosphate buffer pH 6.0 for CelEcc_CBM3a) with 2.5 μL of 10 mM cellotetraose dissolved in ultrapure water and 2.5 μL of either ultrapure water, 70 mM vanillin, or 70 mM tannic acid. Reactions were mixed by vortexing and were run in 0.2 mL PCR tubes for 20 min at 700 rpm and 37 °C. After 20 min, a 2 μL aliquot of the reaction mixture was quenched into a separate 0.2 mL PCR tube containing the oxime tagging solution: 6 μL of 100 mM glycine buffer, pH 1.2, 1 μL of an aqueous solution containing 2.5 mM of [*U*]-^13^C glucose and 2.5 mM of [*U*]-^13^C cellobiose, , 2 μL of CH_3_CN, 1 μL of MeOH, 1.7 μL of NIMS probe (50 mM in 1:1 (v/v) H_2_O:MeOH), and 0.14 μL of aniline. The mixture was incubated at room temperature for 22 h prior to prior to direct spotting on a NIMS chip and subsequent mass spectrometry analysis. All reactions were run in triplicate.

Anaerobic experiments were performed in an anaerobic chamber. Ultrapure water, 100 mM sodium acetate buffer pH 4.6, and 100 mM sodium phosphate buffer pH 6 were sparged with nitrogen prior to pumping into the anaerobic chamber. Cellotetraose and Ab were pumped into the chamber as solid samples, and the enzyme solutions CelEcc_CBM3a and Mt were pumped in as concentrated samples without sparging. All enzyme solutions and dry samples were dissolved or diluted using anaerobic ultrapure water. Reactions were run in 0.2 mL PCR tubes for 20 min at 700 rpm and 37 °C for laccases or 60 °C for CelEcc_CBM3a. After 20 min, a 2 μL aliquot of the reaction mixture was quenched into a separate 0.2 mL PCR tube containing the oxime tagging solution, as described above. The mixture was then removed from the chamber and incubated at room temperature for 22 h prior to prior to direct spotting on a NIMS chip and subsequent mass spectrometry analysis. All reactions were run in triplicate.

For 24 h studies, 20 μL of laccase was added to 25 μL of 100 mM sodium acetate buffer pH 4.6 and 5 μL of substrate (10 mM cellotetraose, 10 mM xylotetraose, or 10 mM mannotetraose, dissolved in ultrapure water), for a final concentration of 1 mM substrate. Reactions were performed in 0.6 mL tubes and vortexed prior to sealing with O_2_− permeable membranes (Breathe-Easy sealing membrane, Sigma). Reactions were run in a Thermomixer at 37 °C, 700 rpm. After 24 h, a 2 μL aliquot of the reaction mixture was transferred to a separate 0.2 mL PCR tube containing the oxime tagging solution. The mixture was incubated at room temperature for 22 h prior to prior to direct spotting on a NIMS chip and subsequent mass spectrometry analysis. All reactions were run in triplicates.

All NIMS analyses were performed on NIMS chips, fabricated as described previously^7, 38^ using a Bruker UltrafleXtreme MALDI TOF-TOF mass spectrometer (Bruker Daltonics, Bremen, Germany). Chips were loaded using a modified standard MALDI plate. FlexControl and FlexAnalysis were used for acquisition and data analysis. Spectra were recorded in positive reflector mode with a laser power of 45%. The instruments were calibrated using either the NIMS internal standards (oxime probe, oxime-tagged [*U*]-^13^C glucose, oxime tagged [*U*]-^13^C cellobiose, or oxime-tagged celloteraose) or Anaspec Peptide Calibration mixture 1 (Anaspec, Fremont, CA). Data were acquired by summing up 3000 laser shots in 500 shot steps, sampling 6 regions locations per spot. Sample spots were identified using grids and inscriptions made with a diamond-tip scribe prior to sample deposition.

For data analysis, Two-way ANOVA was used to make comparisons of different experimental conditions shown in Fig. 4. Error bars represent standard deviation.

## Supplementary Information

Supplementary Information.

## Abbreviations

NIMS: Nanostructure-initiator mass spectrometry
GHs: Glycosyl hydrolases
LME: Lignin modifying enzymes
MALDI-TOF: Matrix-assisted laser desorption ionization-time of flight mass spectrometry
ABTS: 2,2'-Azino-bis(3-ethylbenzothiazoline-6-sulfonic acid)
D.I. water: Deionized water
AFEX: Ammonia fiber expansion
*Ab*: *Agaricus bisporus*
*Mt*: *Myceliopthora thermophila*
Ab: Commercial laccase sample from *Agaricus bisporus*
Mt: Commercial laccase sample from *Myceliopthora thermophila*
