# Supplementary material for: A multiplexed nanostructure-initiator mass spectrometry (NIMS) assay for simultaneously detecting glycosyl hydrolase and lignin modifying enzyme activities
Source: Sci Rep. 2021 Jun 3;11:11803. doi: 10.1038/s41598-021-91181-8 (PMC8175421; doi:10.1038/s41598-021-91181-8)
Supplement: Supplementary file 1 — Supplementary Information. [file 41598_2021_91181_MOESM1_ESM.pdf]

Supplementary Information

A multiplexed nanostructure-initiator mass spectrometry (NIMS) assay for simultaneously detecting glycosyl hydrolase and lignin modifying enzyme activities

Nicole Ing[a,b], Kai Deng[a,b], Yan Chen[a,c], Martina Aulitto [a,c], Jennifer W. Gin[a,c], Le Thanh Mai Pham,[a,b] Christopher J. Petzold[a,c], Steve W. Singer[a,c], Benjamin Bowen[c], Kenneth L. Sale[a,b], Blake A. Simmons[a,c], Anup K. Singh[a,b], Paul D. Adams[a,c,d] and Trent R. Northen[a,c]*

*Correspondence: trnorthen@lbl.gov

[a]Joint BioEnergy Institute, Emeryville, CA 94608
[b]Sandia National Laboratories, Livermore, CA 94551
[c]Lawrence Berkeley National Laboratory, Berkeley, CA 94720
[d]University of California, Berkeley, CA 94720

Proteomics data to confirm the presence of laccases and GH enzymes

Supplementary table 1: Laccase Ab

Protein name	Uniprot accession	# of unique peptides for identifying the protein	# of unique spectra count of identified protein	Protein coverage
Polyphenol oxidase 3	PPO3_AGABI	4	5	5.6%
Polyphenol oxidase 4	PPO4_AGABI	16	23	32%
glucoamylase	K5XZA3_AGABU	7	9	20%
Glycol_hydro_79C domain containing protein	K5WTZ9_AGABU	3	3	8.7%

Supplementary table 2: Laccase Mt

Protein name	Uniprot accession	# of unique peptides for identifying the protein	# of unique spectra count of identified protein	Protein coverage
laccase-2	LAC2_TRAVI	17	24	42%
Laccase-1	LAC1_TRAVI	12	24	25%
Laccase-5	LAC5_TRAVI	2	2	7.4%
glucoamylase	AMYG_ASPOR	8	8	15%

Supplementary Figure 1: Hydrolysis of AFEX pretreated switchgrass by Ab and Mt

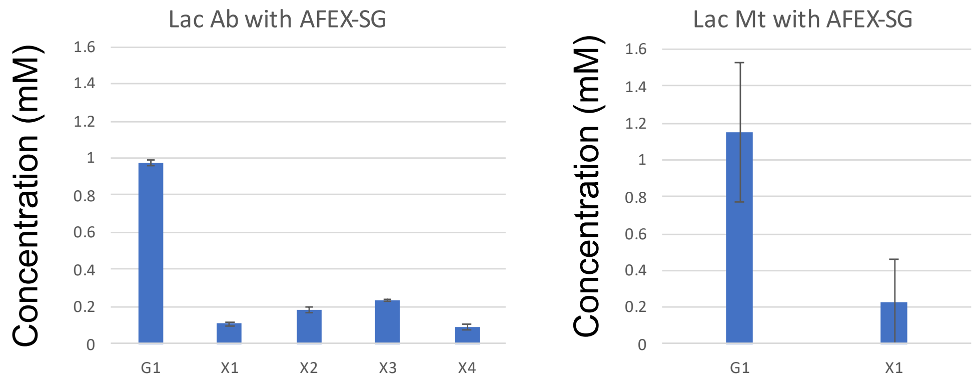

G1=glucose; X1=xylose; X2=xylobiose; X3=xylotriose; X4=xylotetraose
